# Experiences of treadmill walking with non-immersive virtual reality after stroke or acquired brain injury – A qualitative study

**DOI:** 10.1371/journal.pone.0209214

**Published:** 2018-12-14

**Authors:** Karin Törnbom, Anna Danielsson

**Affiliations:** 1 Research group for Rehabilitation Medicine, Institute of Neuroscience and Physiology, Sahlgrenska Academy, University of Gothenburg, Gothenburg, Sweden; 2 Centre for Person-Centred Care, University of Gothenburg, Gothenburg, Sweden; 3 Unit of physiotherapy, Department of Health and Rehabilitation, Institute of Neuroscience and Physiology, Sahlgrenska Academy, University of Gothenburg, Gothenburg, Sweden; University of Central Lancashire, UNITED KINGDOM

## Abstract

**Objectives:**

It is well known that physical activity levels for persons after stroke or acquired brain injuries do not reach existing recommendations. Walking training is highly important since the ability to walk is considered to be a meaningful occupation for most people, and is often reduced after a brain injury. This suggests a need to innovate stroke rehabilitation, so that forms of walking training that are user-friendly and enjoyable can be provided.

**Method:**

An interview study was carried out with persons after stroke (n = 8), or acquired brain injury (n = 2) at a rehabilitation unit at Sahlgrenska University Hospital. We used a semi-structured interview guide to investigate experiences and thoughts about walking on a treadmill with non-immersive virtual reality feedback. The contents were analyzed through an inductive approach, using qualitative content analysis.

**Results:**

The virtual reality experience was perceived as enjoyable, exciting, and challenging. Participants stressed that the visual and auditory feedback increased their motivation to walk on a treadmill. However, for some participants, the virtual reality experience was too challenging, and extreme tiredness or fatigue were reported after the walking session.

**Conclusions:**

Participants’ thoughts and experiences indicated that the Virtual Reality walking system could serve as a complement to more traditional forms of walking training. Early after a brain injury, virtual reality could be a way to train the ability to handle individually adapted multisensory input while walking. Obvious benefits were that participants perceived it as engaging and exciting.

## Introduction

In general, physical activity levels in rehabilitation units are low [1] and do not reach the recommendations for persons with stroke or acquired brain injury (ABI) [2]. There are also indications that the intensity of physiotherapy sessions after stroke is mostly at low levels [3]. Several barriers may contribute to inactivity, such as neurological deficits, cognitive impairment, environmental factors, and lack of motivation [4, 5].

A dose-response effect on exercise outcome after stroke has been shown, and training should be highly repetitive and task oriented [6]. Walking training is important and considered to be a meaningful occupation for most people. To increase walking exercise intensity, treadmill walking has been proposed as a means of task-oriented training that gives the opportunity for many repetitions, and has shown to promote a more normal walking pattern [7]. Walking on a moving surface like a treadmill is more demanding than walking on the ground in terms of sensory processing, postural control and movement coordination. From a motivational perspective, treadmill walking may be perceived as boring the long run.

Training of goal-specific activities with a high number of repetitions may be offered using virtual reality (VR) applications, which have been introduced in neurological rehabilitation [8, 9]. Training using VR has also been suggested to enhance neuroplasticity after stroke [9] by means of offering multisensory stimulation at a high intensity. VR comprises computer-based real-time simulation of an environment with user interaction [10] visually displayed on a screen or through head-mounted devices. Differences in technology and visual presentations in 2D or 3D enable varying types of feedback, levels of immersion and sense of presence in the virtual environment [9, 11]. VR feedback can be mediated through vision, hearing, touch, movement, or smell. The technique provides performance feedback–both directly experienced and objectively quantified, and may thereby increase exercise motivation, and improve motor performance [12–14].

Following stroke, VR training has been mostly described for the upper limb but also for the lower limb; balance and walking as well as for perceptual/cognitive skills [15]. VR has shown a potential for positive effects on walking and balance abilities, although the number of studies are low and the evidence for its superiority to other methods is low [8, 15, 16].

Although few adverse events from VR training have been described, some participants have reported headache or dizziness [15] and knowledge is lacking regarding how persons affected by brain injuries perceive the exposure of multisensory input, during a complex activity such as treadmill walking with VR. The potential effects on motivation and participant experience of VR are scarcely investigated [16] and mostly focused on upper limb activities and games [17]. Based on this, we wanted to investigate patients’ overall experiences of a VR concept in walking training.

The aim of the present study was to explore the experiences of VR in addition to walking on a treadmill in persons with stroke or acquired brain injuries. Participants’ overall experiences and suggestions for development of the exercise method were areas of interest.

## Material and methods

With the incentive to increase motivation for treadmill walking in neurological rehabilitation, ideas using VR were conceptualized and shared by an artist who developed a non-immersive VR system, in collaboration with a physical therapist. An additional incentive was to create a low cost system that would be affordable for rehabilitation units.

### Participants

Patients in the sub-acute stage of stroke, undergoing in—or outpatient rehabilitation at the Sahlgrenska University Hospital were asked to participate. Inclusion criteria were: 1) >18 years of age, 2) stroke or acquired brain injury, 3) impaired walking ability expressed as balance, endurance or coordination problems needing training as defined by their responsible clinical physical therapist, 4) ability to walk on a treadmill with or without physical support, 5) communicative and cognitive ability sufficient to participate in a qualitative interview.

Patients meeting inclusion criteria were identified by their responsible clinical physical therapists. They were informed and asked face-to-face if they wanted to participate in the current study. All patients who were asked agreed to participate. We aimed for a group of participants with diversity regarding sex, age, symptoms, and functional levels. This resulted in a purposive sample of three women and seven men with median 51 years of age (range 38–64). Eight had a stroke diagnosis, one had a traumatic brain injury, and one had had encephalitis. Patients included are hereafter referred to as participants. Neither of the researchers had any prior personal or professional relationship to the participants.

All participants had some degree of walking impairment, e.g. an asymmetric walking pattern or balance problems, although the majority could walk independently over ground. Two persons had pronounced hemiparesis and walked very slowly with aids. On the treadmill, all used the handlebar for support to keep their balance and all walked slower than over ground. All participants were considered by their responsible clinical physiotherapist to be cognitively well functioning in a training and interview situation, although they reported varying levels of fatigue.

For descriptive purposes, motor function was evaluated using the Fugl-Meyer Sensorimotor Assessment Lower extremity section (0–34), where a higher score corresponds to better motor function [18] and Self-selected Over ground Walking Speed was measured using a 30 meter walk test [19]. The participants’ demographic and clinical characteristics are shown in Table 1.

**Table 1:** Demographic and clinical characteristics of the participants (n = 10).

Sex	Age, years	Diagnosis	Time since onset, months	FMA-LE score	Walking aid	Over ground Walking Speed, m/s	Treadmill Speed, m/s
Male	53	Stroke	6	34	None	1.28	0.58
Male	45	Encephalitis	2	34	None	1.11	0.58
Male	38	TBI	5	34	None	1.56	0.72
Female	41	Stroke	7	34	None	1.36	1.22
Male	64	Stroke	3	34	None	1.36	0.42
Male	51	Stroke	6	21	Stick	1.07	0.72
Male	50	Stroke	4	20	Stick, AFO	0.58	0.31
Female	53	Stroke	2	34	None	1.25	0.69
Male	62	Stroke	8	32	None	1.29	0.69
Female	42	Stroke	10	18	Stick, AFO	0.68	0.39

TBI, Traumatic Brain Injury; FMA-LE, Fugl-Meyer Assessment Lower Extremity motor score (0–34); AFO, Ankle Foot Orthosis

### Equipment

A custom-made, non-immersive, 2D VR system based on a video display providing visual and auditory feedback was used. The system being made of off-the-shelf available components was used in combination with a motorized, flat-belt treadmill, sized 0.5m x 1.6m. Custom designed software processed three dimensional acceleration signals from two motion sensors (Wii Remote) via Bluetooth technology. The sensitivity of the motion sensors could be adjusted to match the participant’s walking speed. The motion sensors were attached by elastic straps to the lower legs and the acceleration signals produced by the participants stepping speed controlled the speed of the video display. The treadmill allowed walking speeds from 0 to 2 m/sec with step less increments of 0.1m/sec intervals. Speed was adjusted by the study physiotherapist using the treadmill speed control button on demand by the participant. A flat 92x50 cm screen was mounted 70 cm away from the participant, allowing approximately 70° horizontal field of view. On the screen a 2D video from a real environment was displayed. The video was recorded using a Steadicam held at chest level, while walking on a path in a forest, by the sea, on a road, and in a park. The video was recorded with wide angle perspective at 24 frames/s and a resolution of 720 x460 pixels. Simultaneously, sounds of the participant’s actual step rhythm in addition to nature sounds from the video was mediated through headphones. Walking speed, distance, number of steps and time elapsed were shown to the participant after a walk. To shut out disturbing visual input, the screen and the participant were surrounded by black curtains. Use of an adjustable handlebar mounted in front of the participant was allowed. A physiotherapist stood nearby the participant ready to give support or stop the treadmill. However, there was only one occasion of intervention from the physiotherapist.

### Design and procedure

The study had an inductive qualitative design and criteria for reporting qualitative research, the COREQ-checklist was followed.

At the rehabilitation unit, two walking sessions on the treadmill were undertaken, one with the VR system and one without, in randomized order on two separate days. Walking sessions were conducted by the second author (AD), a female physical therapist, PhD in Rehabilitation Medicine with broad experience from clinical neurological rehabilitation and stroke research. The walking sessions were limited to 30 minutes and the participants stopped walking after a median of 22 (range 13–30) minutes. After each walking session, the participant was asked to rate the exertion on the 6–20 Borg scale Rating of Perceived Exertion [20], and a median of 13 (range 9–18) was rated, indicating a “somewhat strenuous” perception. Spontaneous comments were encouraged and noted by the physical therapist.

Following the second walking session, an individual, face-to-face, semi-structured interview was carried out by the first author (KT), a female MSc in Social Sciences with deep experience in qualitative interview technique and stroke research. Interviews were held at the rehabilitation unit in a quiet room without disturbances.

The interview guide (S1 Appendix) was developed according to the model for conducting qualitative interviews as described by Kvale [21]. The guide covered different aspects of how the VR walking experience was perceived. It also included questions about comparisons between exercising with and without VR. The guide was designed in accordance with physiotherapeutic expertise about cognitive and physical consequences after stroke or other acquired brain injuries. The content of the interview guide was partly derived from prior training of patients in pilot studies with the same VR walking system [22]. The questions were open-ended and the participants were encouraged to speak freely and express their own thoughts about the contents of the interview guide. Depending on answers, exploratory or clarifying follow-up questions were given when needed. In case of new aspects being brought up by the participants, questions were added to the interview guide. The interviews had a mean length of 36 minutes (range 19–61) and were audio-recorded. After ten interviews very little new information was brought up and therefore the material was considered saturated [23].

### Analysis

An inductive, qualitative content analysis as described by Graneheim and Lundman [23] was carried out in collaboration between the two authors. The interviews were transcribed verbatim and then read several times by both authors individually to obtain insight and a general understanding of the studied phenomena. After this, the text was searched for meaning units related to the study questions, and these were highlighted separately by each of the two authors. The meaning units were thereafter summarized and condensed into codes. All codes were compared regarding similarities and differences, and accordingly sorted and collated into categories. To present clear and substantial results, all small categories were merged into main categories. The main categories were then analyzed for underlying, latent meanings and finally themes were formulated. All themes were compared with the initial interview data. Focus continuously moved from the whole to parts of the text to ensure validity of the themes in relation to the entire data set. Throughout the analysis process the two authors discussed and reflected on the findings together. Categories and themes were refined in dialogue between both authors. When disagreement occurred, the matter was discussed until consensus was reached. Examples of the coding process are provided in Table 2.

**Table 2:** Examples illustrating the coding procedure.

Meaning units	Condensed meaning units	Category	Theme
“It was harder (with VR feedback). I had to hold on the whole time because it was moving and I got really tired. I even took off my headphones, because I couldn’t handle all the sounds. I don’t like too much information and talking at the same time, it got to be too much”	“Harder (with VR). I got really tired. Took of headphones because of too much noise. Don’t like too much information at the same time.”	Were not able to handle a lot of input. Workout with VR was experienced as difficult	Exhausting because of too much input
“The bird sounds gave another sense of really being present… and even the footsteps, even if they weren’t exactly synched. Otherwise, I would have wanted to put in more senses! You could have a fan that blows, little breezes on your face as you walk…and smell if you go past a lilac hedge, that would have been lovely”	“Sounds gave a sense of being present. I want to put in more senses! Breezes on your face as you walk, and smell, that had been lovely”	Requests of additional sensory input, that aims to make the VR experience more authentic	Suggestions to improve the VR experience

### Research ethics

The study was approved by the Regional Ethical Review Board in Gothenburg, registration number 060–12. All participants gave written informed consent prior inclusion. Data collection was conducted in accordance with the Declaration of Helsinki.

## Results

Through the inductive qualitative content analysis, three main themes were formulated: 1) *An exciting and enjoyable experience*, 2) *Exhausting because of too much input*, 3) *Suggestions to improve the VR experience*. The themes are illustrated with quotes from the participants, and the numbers do not correlate with the participants’ position in Table 1.

### An exciting and enjoyable experience

Most participants in this study enjoyed the overall VR experience. The video, along with the sounds, was reported as an engaging and exciting way to exercise. Walking on an ordinary treadmill was experienced as somewhat boring, and walking with the VR experience was not. Participants stressed that the visual and auditory feedback made them more willing to walk. Recognition from seeing the same film several times made it possible to know how far one had walked and how far that was left. Reaching the goal faster was reported as an opportunity to compete with oneself.

You wanted to see what was around the next corner. It added a dimension… And I felt like time went faster when you had something to look at, more changing scenery (9).

It was more entertaining. The half an hour went a bit faster than without it. And when you’ve gone on a track for a while and it ends, then something new comes along. It was good (3).

Participants indicated that they valued the sense of being outdoors, in beautiful scenery, without any fear of falling or having to walk on uneven ground. The sense of walking in nature was associated with a feeling of being healthy, and brought back memories of what it had been like walking outdoors before their brain injury.

It is easier to bring your memory back, for a person who had a stroke. Because, you know, some people they used to go to the sea, and some people liked to sit in the forest. This helps them to go back to their memories (7).

Yes, it was a dream location, being out in nature…listening to water flowing and birdsong again. And the greenery, that was a lovely feeling. Peaceful (2).

It was discussed that the video helped participants to walk with improved posture, because it was not possible to look down at their feet and look at the screen simultaneously. It was mentioned that the feeling of walking both downhill and uphill made them use more muscle groups, compared to when just walking straight ahead. Some participants reported that their walking felt steadier and that their pace became more even when walking on the treadmill.

It’s obviously positive exercise, because you walk differently, and use your legs and muscles completely differently. All of a sudden there’s a small decline and you tense your muscles in a different way, compared to just walking normally. It was a good workout for many muscles… (1).

### Exhausting because of too much input

In comparison with walking on an ordinary treadmill, participants were more tired after walking with the VR system. They reported that the VR experience required increased attention and concentration because of the video and the auditory feedback, and some had to focus hard not to lose their balance.

It was harder (with VR feedback). I had to hold on the whole time because it was moving and I got really tired. I even took off my headphones, because I couldn’t handle all the sounds. I don’t like too much information and talking at the same time, it got to be too much (4).

Some participants found the VR experience extremely challenging or even unpleasant because they needed to manage walking straight at a steady pace as well as managing many different kinds of input. Several reported increased fatigue and that they had to rest for hours after the experience. Two participants felt so nauseous that they ended the VR experience before the video had finished.

To start with, it was too exhausting and I was nearly seasick… I don’t think my head can cope with it, if I can say that (2).

It was unpleasant. And really hard work for my brain…sweaty, not because you’d worked hard, but more nerves, and then I started to feel sick and all that… (1)

Participants who felt really disturbed by the audio–visual feedback, said that they preferred to walk on an ordinary treadmill. This would give them a more quiet and controlled experience, with no surprises, and without making them so tired from all the input. However, it was also discussed that letting their brain work hard, with both auditory and visual stimuli, could possibly improve their ability to perceive and interpret various types of input in real life.

If it’s easier next time, then it’s probably a good way to exercise my brain. I don’t know if you can exercise and improve your brain … but if you can, then this ought to be good, because it (my brain) really had to work (4).

Despite the feelings of discomfort that were brought up, all participants were willing to try the VR system again. However, some explained that they needed to feel stronger and be more rehabilitated before trying the VR experience again.

I thought it was a good thing, if you ignore my difficulties. The time passes quickly and you can enjoy the landscape and there are things happening and sounds, that was really good. But right now, I feel like it was unpleasant … I really want to see if I can get better first (1).

I am just a little too sensitive for it right now… when there’s a lot happening at the same time. It’s draining. You get too mentally tired, when there is so much input (10).

Other participants asked for technical adaptations to the VR system, so that it would be better suited for their personal abilities and preferences.

I would like to have the screen dark and just listen to the sounds … but I get a headache from this headband, so I’d want to have just a normal one that you just put over your ears (2).

I could imagine in a calmer environment… it’s this movement. Maybe if it kinda goes in slow motion, because then my brain could keep up better, because then I could focus, or my brain can keep up a little better. And maybe if I get a bit stronger, then I could think about it (going on the treadmill again) (1).

### Suggestions to improve the VR experience

Participants discussed how the VR system could be adjusted into a more appealing way to exercise. Their suggestions were mainly about improvements to the sound effects. Participants wanted the feedback of their footstep to be better in sync, and to change according to what ground they were walking on. They also wanted the birdsong to be equally spread out in the video. The sound from the treadmill was described as disturbing and too loud.

The sound from the motor broke through, so you didn’t hear the surroundings and the bird noises as well (as you should) (9).

It was also suggested that it should be more tiring to walk uphill and easier to walk downhill.

It would have been good if when you go uphill that the treadmill is also doing that. And when it goes downhill, that it feels like downhill … So if it’s uphill, it’s a little bit harder than if it’s flat (4).

Other remarks about the VR experience had to do with safety or comfort. For example, some wanted the movement sensors to be steadier, and the hand support placed on the sides. Participants who felt less disturbed by the amount of sensory information asked for a greater variety of VR feedback. To make the experience more realistic, appropriate smells, air-vents and more natural sounds were suggested. It was suggested that the video should include a dog that was walked on a leash.

The bird sounds gave another sense of really being present… and even the footsteps, even if they weren’t exactly synched. Otherwise, I would have wanted to put in more senses! You could have a fan that blows, little breezes on your face as you walk…and smell if you go past a lilac hedge, that would have been lovely (9).

Several participants reflected on the possibility to choose from a variety of videos, depending on taste and mood. They thought that a greater variety of videos to choose from would increase their motivation to exercise on the treadmill. For example, they wanted to walk on a lovely beach, in an exciting city, or through a museum. Even mountain climbing was suggested.

It’s a bit like the stuff I try and experience when I’m out and walking, have some more exciting environments… I want to explore the surroundings (5).

It would have been more fun with more films to choose from, more variety. I would have like to walk through a castle or a museum (6).

Others wanted to watch the same video every time they exercised, so that they would easily know how long they had walked for. Participants who were disturbed by the amount of input said that the video would be easier to cope with after having watched it several times.

## Discussion

The non-immersive VR walking system used in the present study was developed as a concept to give users a stimulating and varied form of training in a secure and standardized setting. In the present study, we conducted qualitative interviews to explore the experiences of VR in addition to walking on a treadmill, in persons with stroke or ABI.

The most important finding of the study might be that walking with VR was an enjoyable experience. Similar to previous research [24] participants described the experience as entertaining, exciting, challenging, and enjoyable. Although, experiences of training to walk with VR are scarcely investigated, a recent study [25] showed that patients preferred walking with, rather than without VR. That study also showed that patients with VR were less likely to drop-out over a period of four weeks, compared to the group without VR [25].

It has been discussed previously [17, 26, 27] that, because of the feelings that VR often evokes, it provides an experience that is related to that of flow. The feeling of flow [27, 28], mainly comprises concentration, clear goals, positive reinforcement, sense of control, balancing skills and challenges, and losing track of time. In our study, most participants expressed that they were very concentrated during the VR walking session, and a few mentioned that they even lost track of time. Enabling participants to feel distracted in an enjoyable way, while training physically and mentally, was one of the main goals when developing the VR walking system.

A surprising finding in the present study was that some participants expressed strong ambivalent feelings regarding the VR experience. It was experienced as highly enjoyable and at the same time extremely tiring and challenging. Some participants had a positive attitude towards the concept itself but found that the amount of stimuli was too demanding in an early stage of rehabilitation.

The VR system used in this study required some physical strength, an ability to walk on a moving surface without losing balance in addition to concentrating on audio and visual input simultaneously. The physical capacity and, thereby, the perceived level of physical effort probably differed between participants. In addition, when having to concentrate on several sensory inputs, almost all participants—even those with a higher physical capacity, experienced fatigue to some extent. Participants’ statements concerning fatigue and tiredness showed that the cognitive level of functioning seemed to be central for how walking with VR was experienced. It also seemed that physical and cognitive challenges were related to each other, and were somewhat difficult to distinguish. For example, how much of the tiredness came from concentrating on holding their balance and trying to walk straight? And, to what extent did participants get tired from the effort to cope with audio—visual input? Several participants stated that they couldn’t manage all these components at the same time; and that was in line with previous research [17, 29].

Dizziness or sickness reported by some of the participants were probably related to a conflict between visual, vestibular and proprioceptive input, known as motion or simulator sickness which is a well-known phenomenon induced by VR [30]. The visual presentation that was provided by the present application, in combination with a moving walking surface, could contribute to this sensory conflict. The present VR system provided auditory feedback on walking speed by step sounds reflecting the participant’s actual step rhythm. The importance of realistic and well synchronized auditory step feedback was emphasized by some participants. If steps made are not well synchronized with steps heard, participants will probably become extra tired from coping with this discrepancy. Sensory feedback, such as the footsteps sounds, given by auditory input has been shown to have a role in motor learning. Effects on walking speed and stride length have been seen with auditory rhythmic cueing at a set pace [31, 32], but auditory feedback on step rhythm may also increase walking speed, as was shown in a study of children with cerebral palsy [33].

The participants who experienced the VR walking as challenging also stated that it was difficult and less suitable for them in their current stage of rehabilitation. We suggest that the technique should make it possible to disconnect audio–or visual feedback if the user wants to. It is also possible that the user would feel less tired from concentrating on keeping their balance if they got support from a harness. However, participants in the present study mentioned that the VR walking served as valuable mental exercise in coping with simultaneous and varied input. In previous research [9], it has been suggested that multisensory information may challenge sensorimotor function in a positive way. According to a systematic review on upper-limb virtual reality after stroke [34] VR can contribute to cognitive rehabilitation. A similar study suggested that walking training with additional cognitive or motor tasks can improve mobility [35]. In addition, combined motor and cognitive training using VR can also decrease the risk of falls [36].

It has also been shown earlier [37] that lower fatigue was reported by stroke patients using VR-based motor training. Therefore, the VR experience could possibly be a suitable tool in preparing persons with brain injuries for dual tasking in real-life situations [26], both by improving their ability to handle several inputs at the same time, and to serve as an inspiration for rehabilitation. This type of VR training could therefore also work as a form of assessment of a person’s capacity to process multisensory input.

In contrast to previous research [17, 24, 28] the VR system in this study did not include a dimension of competitiveness as it was not designed to be a game. However, in addition to the continuous visual and auditory feedback given during walking, participants could compete with themselves in terms of walking speed and endurance, which was also emphasized by some participants. Previous research [38] showed that being able to measure one’s progress or relapse in a rehabilitation program are important to increase the participants motivation to do more. This aspect, together with the knowledge of how clear goals and positive reinforcement can contribute to a feeling of flow should be taken into account when creating a VR walking system for stroke rehabilitation [24].

A Danish study [17] found that older participants preferred using more traditional approaches to therapy and that it was difficult to get them interested in training with VR. In the present study group participants were younger compared to an average stroke population and everyone was motivated to try the VR walking. The VR walking system was also user-friendly. Even though participants were unfamiliar with the VR technology *per se*, they were familiar with walking on a treadmill, and all of them liked the beautiful sea and rural settings of the video. Participants were pleasantly surprised that the film brought back memories of walking in nature. In addition, walking training was considered a meaningful activity, and all participants wanted to improve their walking ability.

Participants that did not feel so cognitively challenged by the VR experience asked if it was possible to add input, e.g., to feel the wind blowing, or to make the audio -visual feedback more accurate in terms of timing or type of sounds. It is possible that participants wanted to make the experience more engaging, by ensuring a rich and lifelike sensory feedback. In addition, participants speculated freely and gave several suggestions about types of videos that they would like to add to the VR experience. This was also in the mind of the artist who conceptualized the idea of the current VR system. The enthusiasm and interest that was shown suggested that participants really enjoyed engaging in this form of physical training. We share the idea that videos from various environments, that meets different needs and interests, would make the VR walking system even more attractive to use. In addition, exercising while being exposed to cognitive challenges, with an appropriate level of difficulty is important for an optimal recovery [6].

In this early stage a controlled and supervised setting of VR training is probably needed. However, in later stages after brain injury, the VR walking system could be used in a home environment, enabling persons to fit their rehabilitation routine in with everyday life, and enabling them to use it more frequently. The VR equipment used in the present study was a relatively simple 2D, non-immersive system. In contrast to more sophisticated VR systems developed for walking training, offering more dimensions, a low cost system like the present one could be affordable, and thereby enable a form of enriched milieu in various rehabilitation settings [39]. It is also possible that a refined VR system, providing more sophisticated feedback, e.g., better synchronized step sounds, would be experienced as less tiring.

### Strengths and limitations

The qualitative design made the results rich and the content was not limited by the design of a questionnaire. The design made it also possible to examine the research topic in depth. Another strength of this study was that a range in participants’ functional status widened the variation in experiences and gave a broad picture of the study phenomena. Participants were considered representative for the rehabilitation clinic regarding sex distribution, age span and mix of the two diagnoses stroke versus ABI. Having two diagnoses in the same study might however make interpretation of the results more difficult. Since a part of the results focused on experienced poor cognitive functions, a limitation was that we did not include a measurement of cognitive ability in the study. We believe that central issues were covered in the interview guide as physiotherapeutic expertise in training patients with brain injuries was considered. In addition, prior experience from the developmental process and a pilot study of training with the VR-system [22] contributed to the questions posed. However, it cannot be excluded that bias may have had impact on interview guide content or affected the data analysis and interpretation of results.

## Conclusions

It is well known that physical activity levels for persons after stroke or ABI do not reach recommended levels [2]. This suggests a need to innovate neurological rehabilitation and to provide a variety of forms of physical training that are user-friendly and enjoyable.

Participants’ thoughts and reasoning indicated that the VR walking system could serve as a complement to more traditional forms of walking training. The most obvious benefits were that participants perceived it as engaging and exciting.

It was clear that the VR walking system needed some adjustments to tailor it for persons with different levels of physical and cognitive functioning. Receiving a lot of input was experienced as cognitively tiring. However, it seemed that the benefits compensated for the negative experiences to some extent, since even participants who found the VR experience somewhat unpleasant wanted to try it again.

The VR technology for walking training in stroke rehabilitation can be seen as a promising part of the work to provide high-intensity task-specific exercise although its potential to serve as a means to increase physical activity has to be investigated. Future studies should also address the suitability of VR training methods for patients with different conditions and stages after injury. With advancement in technology, more sophisticated VR equipment is available at reasonable costs. Thus, it is possible to construct future studies with more optimal VR walking experiences.

## Supporting information

S1 Appendix Interview guide. (DOCX)
