# Supplementary material for: Experiences of treadmill walking with non-immersive virtual reality after stroke or acquired brain injury – A qualitative study
Source: PLoS One. 2018 Dec 14;13(12):e0209214. doi: 10.1371/journal.pone.0209214 (PMC6294388; doi:10.1371/journal.pone.0209214)
Supplement: S1 Appendix — (DOCX) [file pone.0209214.s001.docx]

**Interview guide for exercising on a treadmill with VR**

What was walking on a treadmill like?

How did walking on a treadmill differ from other walking exercise/training?

What did you like about walking on the treadmill with the VR system?

What did you think? How was it different?

How did it compare to other types of exercise/training?

What did seeing the film feel like?

What did you think of the environment?

What did you think about the sounds that accompanied the film?

What did you like and what did you not like? Can you explain why?

Was any of it uncomfortable? Can you explain why?

What did you think of the equipment?

How well did you think the controls for the treadmill worked? Could you walk at the pace you wanted?

Did you use the side supports to steady yourself?

How well do you think the controls for the film speed worked?

What did you think of the film?

Did you feel any difference between walking on the treadmill with versus without VR?

Would you like to improve the treadmill or the VR experience in any way?

How did you think these improvements would impact your experience of exercising/training with VR?
